# Is C-reactive protein the answer to antibiotics over-prescribing in primary care? A behavioural experiment in two European countries

**DOI:** 10.1093/jacamr/dlag209

**Published:** 2026-09-17

**Authors:** Martine Nurek, Alastair D Hay, Peder A Halvorsen, Olga Kostopoulou

**Affiliations:** Department of Surgery and Cancer, Faculty of Medicine, Imperial College London, St Mary’s Campus, Paterson Building, S Wharf Road, London W2 1NY, UK; Centre for Academic Primary Care, Bristol Medical School: Population Health Sciences, University of Bristol, Canynge Hall, 39 Whatley Road, Bristol BS8 2PS, UK; Department of Community Medicine, Faculty of Health Sciences, The Arctic University of Norway, PO Box 6060 Stakkevollan, N-9037 Tromsø, Norway; Department of Surgery and Cancer, Faculty of Medicine, Imperial College London, St Mary’s Campus, Paterson Building, S Wharf Road, London W2 1NY, UK; Institute of Global Health Innovation, Imperial College London, Level 1, Faculty Building, South Kensington Campus, London SW7 2NA, UK

## Abstract

**Background:**

Antibiotic overprescribing fuels antimicrobial resistance. C-reactive protein (CRP) is widely promoted as a point-of-care test for targeted prescribing. While routinely used in some European countries such as Norway, it is not part of standard practice in UK primary care. Understanding how CRP testing might impact general practitioners’ (GP) judgement is critical for informed implementation. We explored this with UK and Norwegian GPs.

**Methods:**

One hundred and one GPs per country responded to four scenarios, describing patients with a range of infectious or inflammatory conditions. After reading a patient description, GPs could request further information, including CRP. The CRP result was either high or low, randomly assigned per GP and scenario. Each time GPs obtained new information, they indicated whether antibiotics were needed (Likert scale). At the end, they selected a management option.

**Results:**

Norwegian GPs requested CRP more often than UK GPs [77% versus 28%, odds ratio (OR) = 18.09 (95% CI 9.86–33.20), *P* < 0.001], but prescribed antibiotics at similar rates [39% versus 42%, OR = 1.36 (95% CI 0.96–1.94), *P* = 0.085]. When CRP was requested, elevated values increased prescribing odds [OR = 1.84 (95% CI 1.15–2.97), *P* = 0.012], while low values had no impact. If GPs believed antibiotics were needed, a low CRP did not reassure them. Higher uncertainty led to more CRP requests, but these did not reduce uncertainty.

**Conclusions:**

Despite marked differences in CRP requests, we detected no between-country differences in prescribing. Elevated CRP increased prescribing, whereas low CRP was not sufficiently reassuring. The results do not support routine use of CRP as an antimicrobial stewardship tool in primary care.

## Introduction

Antimicrobial resistance is now recognized as one of the most pressing threats to humanity.^1^ A host of behavioural factors contribute to the overuse of antibiotics,^2^ one of them being unnecessary prescribing in primary care.^3,4^ Several interventions have been introduced to curb overprescribing, ranging from training in communication skills,^5^ nudges,^6,7^ clinical prediction scores,^8^ and point-of-care tests (POCTs).^9^ C-reactive protein (CRP) is a POCT that measures the immune response to an infection. It is a blood test that can provide a result in 3–4 min, which can inform decision-making during the consultation. The evidence for its effectiveness is, however, mixed, with some studies strongly supporting its use^10^ and others finding no evidence that it reduces prescribing.^11–13^ A recently updated Cochrane review concluded that ‘C-reactive protein point-of-care tests in children and adults likely reduce the number of participants given an antibiotic prescription for acute respiratory infections in primary care’.^14^ The evidence assessed in the review was considered to be of ‘moderate certainty’.

CRP is now routinely used in primary care in several European countries, including Norway. Norwegian guidelines suggest that CRP may be useful for differentiating between acute bronchitis and pneumonia and for deciding whether to prescribe antibiotics for COPD exacerbations. It is also recommended to be used routinely in children with suspected urinary tract infection (UTI).^15^ In many other European countries, CRP features in the guidelines for suspected respiratory tract infections but has not been incorporated into those for UTIs. In contrast, CRP is not routinely used in the UK and its uptake is low. NICE guidance for the management of lower respiratory infections in adults (2023) suggests that CRP-POCT be considered when clinical assessment is inconclusive.^16^

Given the uncertain evidence, this study aimed to explore the impact of CRP-POCT on clinical judgement. To do this, we ran an online behavioural experiment using clinical scenarios, which depicted different health complaints for which a general practitioner (GP) might consider antibiotics and/or hospitalization. Two scenarios depicted patients with respiratory tract infections, one scenario depicted a patient with possible appendicitis and another scenario depicted a child with a possible UTI. We followed a process-tracing methodology,^17^ whereby participants could request information from a pool of items (including CRP) and provided their judgements at each step. This allowed us to monitor how GPs’ perceived need for antibiotics and/or hospitalization evolved during the scenarios and to measure the timing, frequency and impact of CRP requests. We recruited large samples of GPs from two European countries, Norway and the UK, and expected between-country differences at least in the frequency of CRP requests.

## Materials and methods

### Ethics

The UK arm of the study was approved by the Imperial College Research Ethics Committee, reference number 6323317. The Norwegian arm was exempt under the Norwegian Health Research Act; Sikt, the Norwegian Agency for Shared Services in Education and Research, was notified and confirmed that no formal assessment was needed, as the data were anonymous. The research was conducted in accordance with the principles of the Declaration of Helsinki. All participants provided informed consent online before starting the study.

### Participants

We powered the study to detect a small effect size (*f*^2^ = 0.02) in a two-tailed multiple linear regression predicting perceived need for antibiotics based on CRP status (unknown/low/high) and country (UK/Norway). Using G*Power 3.1, we estimated that 528 responses would be required for 90% power and *α* = 0.05. We then calculated the design effect (DE) expected for a cluster of four responses per participant, using the formula DE = 1 + (*n* – 1)*ρ*,^18,19^ where *n* is the cluster size (four scenarios) and *ρ* is the intraclass correlation, expected to be small (0.05) due to the diversity of the scenarios. Adjusting the previously calculated sample size (528) by the DE (1.15) called for at least 607 responses (528 × 1.15 = 607). At four responses per participant, at least 152 GPs would be required (607 ÷ 4 = 152).

UK and Norwegian GPs with at least a year’s practice experience in their respective countries were eligible to take part. UK GPs were recruited from a database of previous study participants. Norwegian GPs were recruited from practices in the Norwegian Primary Care Research Network (PraksisNett).^20^ Prospective participants were sent an invitation e-mail explaining the study aim and containing a link to an expression-of-interest form. Upon study completion, UK participants received £50 and Norwegians NOK 1180 (approx. £85) via bank transfer. UK data were collected February–March 2023; Norwegian data October 2023–March 2024.

### Design

The experiment followed a 2 (country)×2 (CRP low/high) mixed factorial design. Participants responded to four purpose-built patient scenarios. Each scenario had two versions: one with high CRP (range 75–126 mg/L) and the other with low CRP (range 18–32 mg/L). Scenario versions were otherwise identical and allocated at random (per participant per scenario).

Scenarios were constructed by two clinician authors (A.D.H. and P.A.H.), both experienced GPs and academic researchers. Each scenario began with a patient description comprising demographic details (age, sex, and BMI), relevant medical history, current medications and health complaint (respiratory in two scenarios, abdominal in the other two). A further five to seven information items were available in each scenario upon request: *patient appearance*, *temperature*, *CRP result* (available in all scenarios); *breathing*, *oxygen saturation*, *pulse*, *chest auscultation* (respiratory scenarios only); *abdominal examination*, *urine dip/U-stix* (abdominal scenarios only).

Scenarios were translated to Norwegian by a third-party provider, back-translated by a bilingual academic GP and pilot-tested on 12 GPs (6 UK, 6 Norwegian). The scenarios can be found in Supplement S1 (available as Supplementary data at *JAC-AMR* Online) in both languages.

### Procedure

Eligible GPs who had expressed interest were e-mailed a link to the study website (hosted by Qualtrics), where they read a study information sheet and gave informed consent. They then encountered a practice scenario (abdominal complaint, low CRP value), intended to familiarize them with the study interface, decision task and procedure.

GPs then saw the four scenarios (order randomized per GP). Screenshots illustrating the study procedure can be found in Supplement S2. Based on the initial patient description, GPs rated their agreement with two statements on separate Likert scales ranging from 0 (strongly disagree) to 6 (strongly agree):

- I believe that an antibiotic is necessary to treat this patient’s infection.
- I believe that this patient needs to be admitted to hospital.

We named these variables ‘perceived need for antibiotics’ and ‘perceived need for hospitalization’.

GPs could then gather more information by selecting items from a list (item order randomized per GP). They were instructed to gather only information that they really needed to manage the patient appropriately. They were also told that they would be shown all information at the end, to satisfy their curiosity. When an information item was requested, the answer was presented at the top of a new screen, and GPs were asked to rate again their perceived need for antibiotics and hospitalization. They were also asked whether they had decided on management. If they had not, they were redirected to the list of remaining items to repeat the process, until they had decided how to manage or had viewed all the items. In either case, GPs could select at least one management option (and elaborate in free text if prompted):

- Provide information about symptom duration and safety net
- Arrange a follow-up appointment/review (when?)
- Order investigations (which investigations?)
- Prescribe antibiotics
- Prescribe other medication (what would you prescribe?)
- Refer to a specialist (what kind of specialist?)
- Admit to hospital
- Other (please explain)

Following this, GPs were asked to justify their management decision (compulsory, free-text response) and to provide any diagnostic hypotheses they might have.

After completing all four scenarios, GPs were shown complete versions, including information they had not previously requested. For each complete scenario, they were asked to rate again their perceived need for antibiotics and hospitalization, select management option(s) and justify their decision.

### Construction of variables and analyses

Data were analysed using mixed effects regression models with a random intercept for GP to account for clustered data. Requesting CRP (0 = not requested, 1 = requested) and the timing of CRP requests (1 = first item requested, 2 = second item requested, etc.) were compared between countries (0 = UK, 1 = Norway) using logistic and linear regression, respectively.

‘Time’ indicated when a variable was measured. Time 1 (scenario start) indicated measurements taken after the initial patient description, before a GP started gathering additional information. Time 2 (scenario end) indicated measurements taken at the end of data gathering, before management was selected. Time 3 (scenario review) indicated measurements taken after all the scenario information was revealed.

We constructed a variable called ‘CRP status’ with three categories: 0 = unknown (CRP not requested), 1 = low and 2 = high. To measure the impact of CRP on perceived need for antibiotics and prescribing decisions at Time 2, we built two regression models. The first was a linear model regressing perceived need for antibiotics (rating on the 0–6 scale) on CRP status and country. The second was a binary logistic model regressing antibiotic prescribing decisions (0 = no prescription, 1 = prescription) on CRP status and country. Both models used ‘unknown CRP’ as the reference category and controlled for perceived need for antibiotics at Time 1.

The above analyses were repeated for perceived need for hospitalization (0–6 scale) and decisions to escalate care (0 = no escalation, 1 = escalation). Decisions to escalate care merged two management options: referrals to a specialist and hospital admissions. If either had been selected, the decision to escalate care was coded as 1. The models controlled for perceived need for hospitalization at Time 1 and country.

To explore the relationship between CRP and GP uncertainty, we recoded the perceived need for antibiotics (‘I believe that an antibiotic is necessary to treat this patient’s infection’) as follows: neither agree nor disagree = 0 (uncertain), somewhat agree/disagree = 1 (somewhat certain), agree/disagree = 2 (certain) and strongly agree/disagree = 3 (very certain). We used binary logistic regression to assess whether uncertainty at Time 1 impacted requesting CRP (0/1); and linear regression to assess whether requesting CRP impacted uncertainty at Time 2, controlling for uncertainty at Time 1. Both models included country as a factor.

We determined whether the CRP result was consistent with the GP’s clinical impression up to that point: a CRP result that immediately followed a low perceived need for antibiotics (a rating of ≤2, i.e. somewhat disagree/disagree/strongly disagree) was classified as ‘expected’ if it was low, and ‘unexpected’ if it was high. Similarly, a CRP result that followed a high perceived need for antibiotics (a rating of ≥4, i.e. somewhat agree/agree/strongly agree) was ‘unexpected’ if it was low and ‘expected’ if it was high. We excluded instances where GPs were uncertain about the need for antibiotics prior to receiving the CRP result (perceived need = 3). This returned a four-category variable describing the CRP result as ‘unexpectedly low’ (0), ‘unexpectedly high’ (1), ‘expectedly low’ (2) or ‘expectedly high’ (3). Using linear regression, we measured whether this variable predicted prescribing at Time 2 (0 = no prescription, 1 = prescription), accounting for perceived need at Time 1 and country. We used ‘expectedly low CRP’ as the reference category. We excluded cases where GPs chose to escalate care without prescribing antibiotics. Analyses were conducted using Stata/MP 17.0.

Finally, we analysed thematically all free-text comments that mentioned CRP. These results are presented in Supplement S4.

## Results

We invited 566 GPs: 242 UK and 324 Norwegian. Of the 224 GPs that started the survey, 22 (10%) did not complete it; their data were excluded. The final sample thus comprised 202 GPs: 101 UK and 101 Norwegian. Sample characteristics appear in Table 1.

**Table 1.** Characteristics of the UK and Norwegian GP samples

Sample characteristics		
Gender (female/male/non-binary/prefer not to say)	UK	50% female (51/101); 50% male (50/101)
	Norway	50% female (50/101); 50% male (51/101)
Years since GP qualification (mean, SD)	UK	11.9 (SD 8.1)
	Norway	12.9 (SD 9.8)
Routine use of CRP-POCT^a^ (yes/no)	UK	5% yes (5/98)
	Norway	90% yes (85/95)

^a^At the end of the study, GPs were asked: ‘Do you use point-of-care CRP tests as part of your routine practice?’ 3 UK and 6 Norwegian GPs did not answer this question.

Table 2 presents the results for the outcome measures by country and time. At Time 1, UK GPs perceived higher need for antibiotics than Norwegian GPs [*b* = 0.56 (0.31–0.81), *P* < 0.001] and this persisted throughout. UK GPs prescribed antibiotics and escalated care more frequently but not significantly so [antibiotics: Time 2 odds ratio (OR) = 1.36 (0.96–1.94), *P* = 0.085; Time 3 OR = 0.99 (0.71–1.39), *P* = 0.976; escalation: Time 2 OR = 0.73 (0.51–1.04), *P* = 0.082; Time 3 OR = 0.75 (0.53–1.06), *P* = 0.105].

**Table 2.** Outcome measures by country and time

		Time 1 (scenario start)	Time 2 (scenario end)	Time 3 (scenario review)
Perceived need for antibiotics Mean (SD) median (range)	UK	3.38 (0.86) 4 (0–6)	3.30 (0.91) 4 (0–6)	3.64 (0.88) 5 (0–6)
	Norway	2.82 (0.87) 3 (0–6)	2.96 (1.06) 3 (0–6)	3.05 (0.89) 3 (0–6)
Perceived need for hospitalization Mean (SD) median (range)	UK	1.98 (1.34) 2 (0–6)	2.23 (2.12) 1 (0–6)	2.45 (2.10) 1 (0–6)
	Norway	1.88 (1.49) 2 (0–6)	1.84 (2.05) 1 (0–6)	1.93 (2.12) 1 (0–6)
CRP requests (%, frequency)	UK	—	28% (112)	—
	Norway	—	77% (311)	—
Order of CRP requests Median (range)	UK	—	4 (1–7)	—
	Norway	—	3 (1–7)	—
% Antibiotics prescriptions (frequency)	UK	—	42 (170)	47 (190)
	Norway	—	39 (156)	39 (158)
% Care escalation decisions (frequency)	UK	—	29 (118)	31 (124)
	Norway	—	24 (98)	26 (104)
% Further exploration decisions (frequency)	UK	—	28 (111)	32 (131)
	Norway	—	30 (119)	32 (128)
% Safety netting decisions (frequency)	UK	—	45 (180)	42 (168)
	Norway	—	44 (178)	42 (168)
Other	UK	—	18 (73)	20 (80)
	Norway	—	20 (79)	22 (89)

The denominator for the calculation of the percentages is 404, the total number of scenario presentations. ‘Care escalation’ includes hospitalization and/or referral to specialist services. ‘Further exploration’ includes arranging follow-up appointments and/or ordering investigations. ‘Other’ includes any other form of management, e.g. prescribing other medications.

GPs requested 69%–91% of the information items available, depending on the scenario. Few decisions changed from Time 2 to Time 3: antibiotics prescriptions increased by 5% in the UK sample (no change in the Norwegian sample), while decisions to escalate care increased by 2% in both samples (Table 2). Supplement S3 presents frequency of decisions at Time 2 and Time 3 by scenario, CRP status and country. The scenario designed to depict appendicitis (William) resulted predominantly in care escalation decisions both at Time 2 and Time 3 (Tables S1 and S2).

As expected, Norwegian GPs requested CRP significantly more often than UK GPs [OR = 18.09 (9.86–33.20), *P* < 0.001]. CRP was most commonly the third item requested by Norwegian GPs and the fourth by UK GPs (Table 2).

Perceived need for antibiotics at Time 1 predicted both perceived need for antibiotics at Time 2 [*b* = 0.88 (0.82–0.93), *P* < 0.001] and prescribing decisions [OR = 2.54 (2.22–2.89), *P* < 0.001]. Similarly, perceived need for hospitalization at Time 1 predicted both perceived need at Time 2 [*b* = 0.81 (0.73–0.89), *P* < 0.001] and decisions to escalate care [OR = 2.50 (2.14–2.93), *P* < 0.001].

### Impact of CRP result on perceptions and decisions

**Figure 1.**
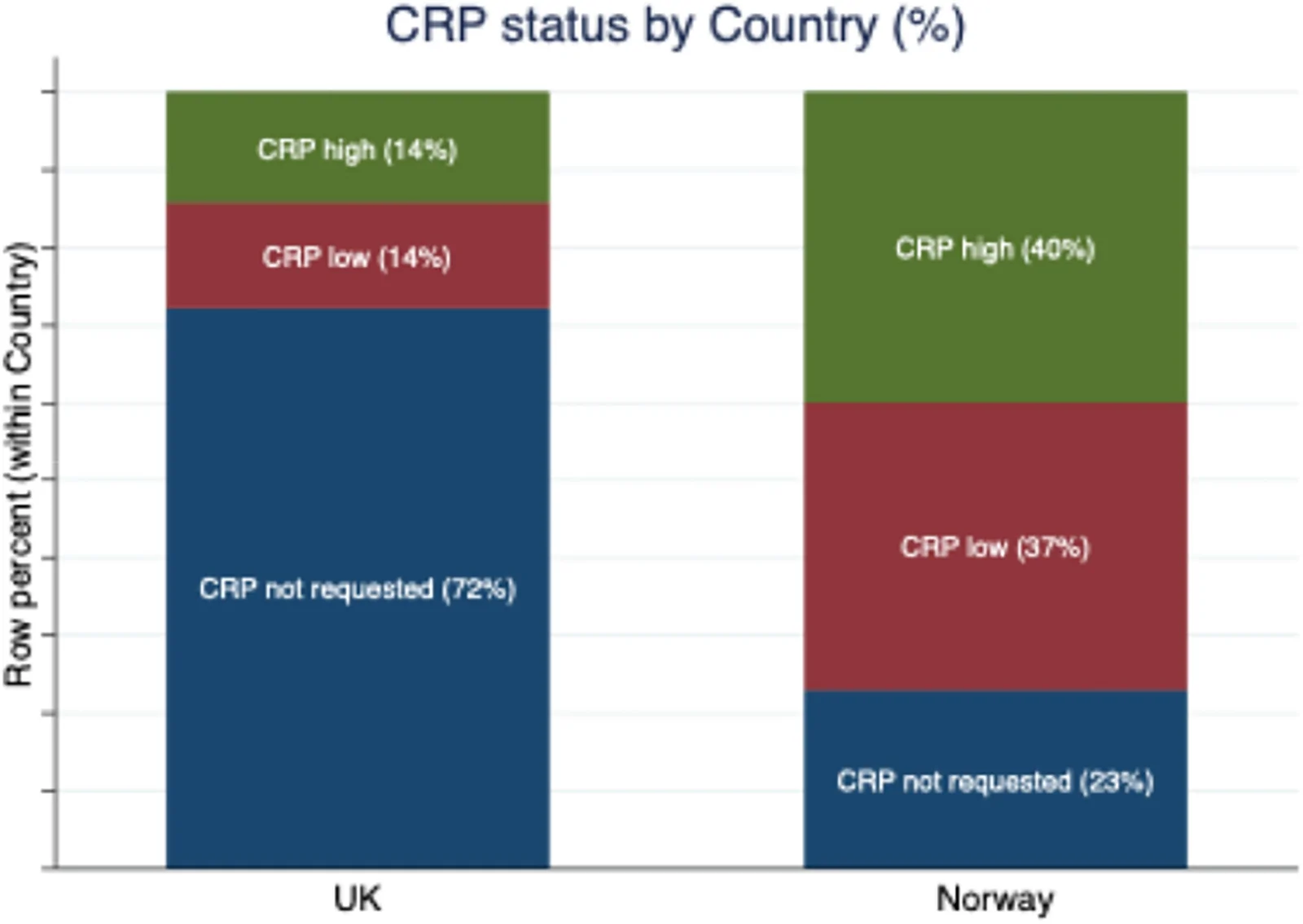
Stacked bar chart showing percentage of instances when CRP was not requested (lower segment), requested and found to be low (middle segment) and requested and found to be high (upper segment), presented separately for the UK and Norwegian samples.

Given large differences in the proportion of unknown, low and high CRP results in the two countries (Figure 1), we conducted the following analyses first for the whole sample, then per country.

**Figure 2.**
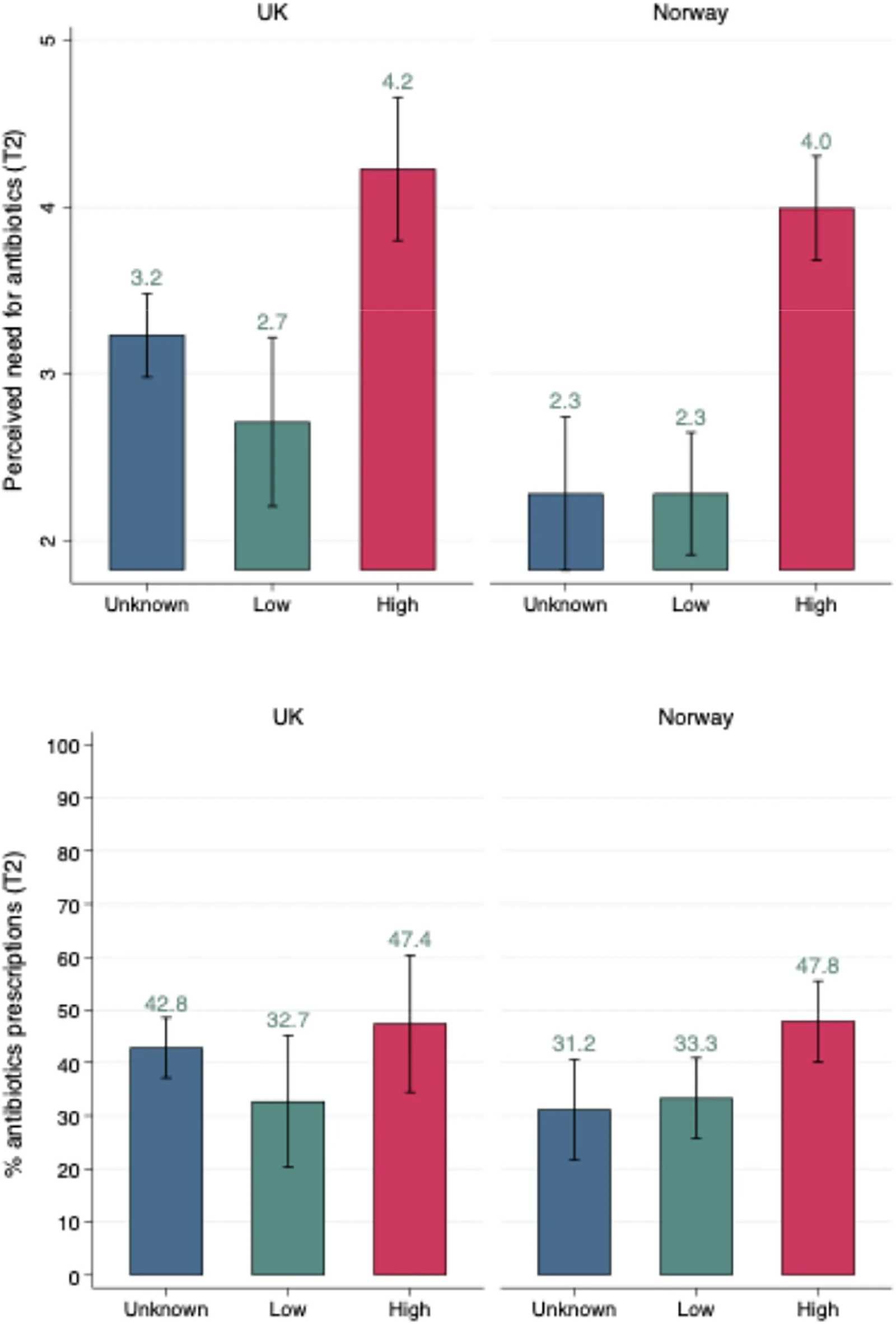
Bar graphs presenting mean perceived need for antibiotics at Time 2 (top panel) and percentage of antibiotic prescribing decisions at Time 2 (bottom panel) with 95% CIs by CRP status and country.

When CRP was requested and found to be high, GPs perceived significantly higher need for antibiotics [*b* = 1.24 (0.97–1.51), *P* < 0.001] and prescribed them more often [OR = 1.84 (1.15–2.97), *P* = 0.012] than when CRP was unknown (not requested; Figure 2). When analyses were conducted per country, only in Norway were prescriptions more frequent with high versus unknown CRP [*b* = 2.57 (1.28–5.16), *P* = 0.008]. GPs also perceived a greater need for hospitalization [*b* = 1.11 (0.80–1.41), *P* < 0.001] and opted to escalate care more often [OR = 2.57 (1.60–4.14), *P* < 0.001] when CRP was high. When analyses were conducted per country, only in the UK were care escalation decisions more frequent with high versus unknown CRP [OR = 3.47 (1.78–6.74), *P* < 0.001].

When CRP was found to be low, GPs perceived somewhat lower need for antibiotics [*b* = −0.30 (−0.57 to 0.03), *P* = 0.031] but did not prescribe them less often than when they had not requested CRP [OR = 0.86 (0.53–1.39), *P* = 0.540]. Analysis per country revealed no significant effects (Figure 2). Neither was there an impact of low CRP on perceived need for hospitalization [*b* = −0.06 (−0.37 to 0.24), *P* = 0.683] or decisions to escalate care [OR = 0.90 (0.54–1.50), *P* = 0.685].

### Consistency between clinical impression (perceived need for antibiotics) and CRP result

**Figure 3.**
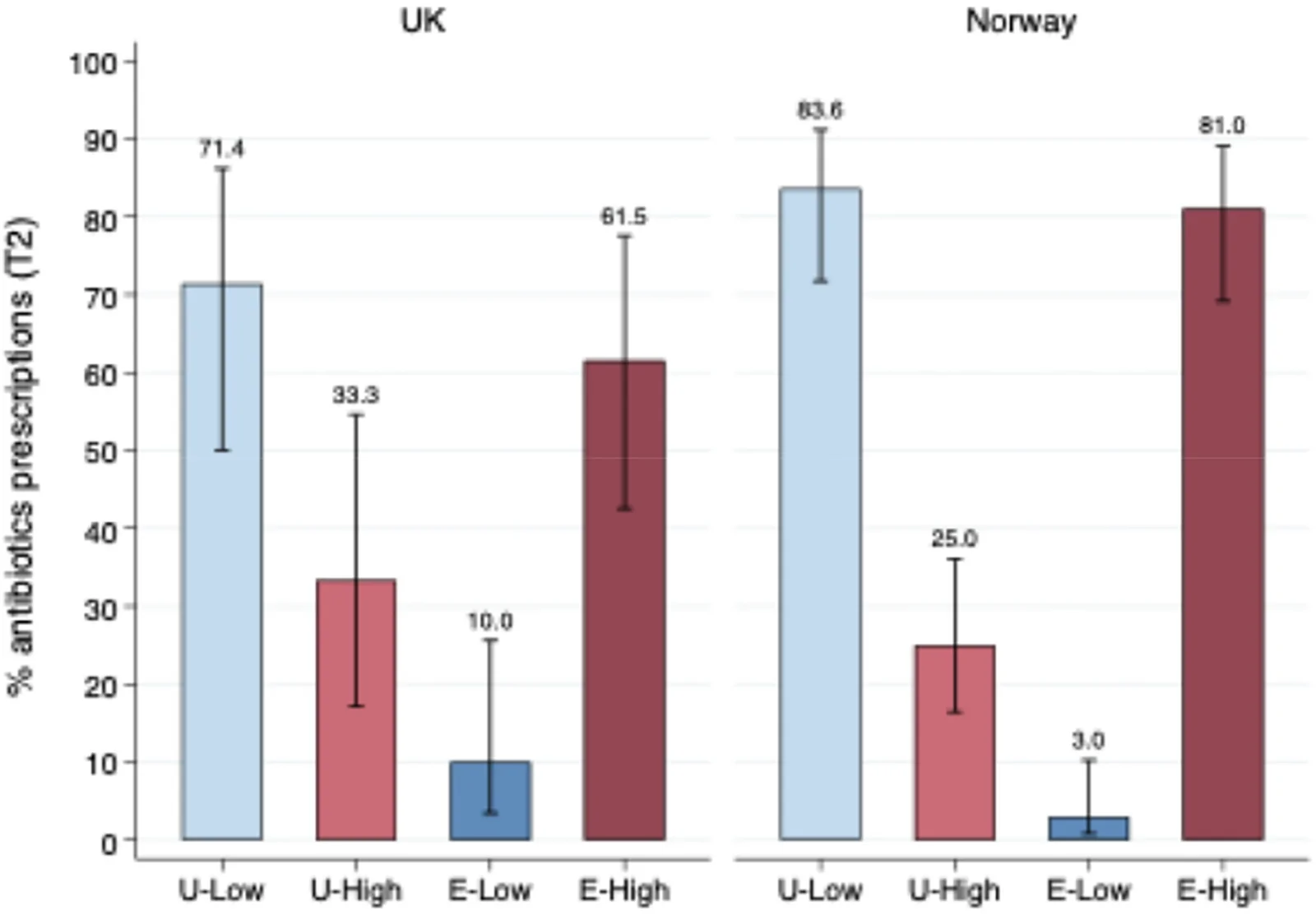
Bar graphs per country presenting percentage of Time 2 antibiotics prescribing decisions according to whether CRP was high or low and expected or unexpected. U-Low, unexpectedly low; U-High, unexpectedly high; E-Low, expectedly low; E-High, expectedly high. Per bar % was calculated across all antibiotics prescribing decisions (i.e. yes = 1 and no = 0) within each consistency category and country. Due to small numbers in some consistency × country combinations, Wilson CIs were used for the error bars.

Compared with GPs who did not think antibiotics were needed and then saw a low CRP value (‘expectedly low CRP’), those who saw an unexpectedly low CRP and also those who saw a high CRP (expected or unexpected) were significantly more likely to prescribe antibiotics: OR = 32.68 (6.74–158.42), *P* < 0.001, for ‘unexpectedly low CRP’; OR = 132.40 (18.20–963.38), *P* < 0.001, for ‘expectedly high CRP’; and OR = 16.49 (4.18–65.08), *P* < 0.001, for ‘unexpectedly high CRP’ (205 instances of escalation-without-antibiotics decisions were excluded from this analysis). When a high CRP confirmed the clinical impression, it moved GPs to prescribe antibiotics more than when it was unexpected (OR = 132.40 versus OR = 16.49; Figure 3, E-High versus U-High). Furthermore, when GPs thought that antibiotics were needed and saw an unexpectedly low CRP, this did not reassure them (Figure 3, E-Low versus U-Low). In both countries, unexpectedly low CRP was associated with the most prescriptions. These results suggest that the clinical impression weighed more heavily on prescribing decisions than an unexpected CRP result.

### CRP and certainty

Greater initial certainty about whether antibiotics were needed reduced the odds of requesting CRP [OR = 0.68 (0.55–0.83), *P* < 0.001]. Obtaining a CRP result however did not impact certainty [*b* = −0.07 (−0.19 to 0.05), *P* = 0.253)—unless it was unexpected, which reduced certainty [*b* = −0.34 (−0.49 to −0.20], *P* < 0.001]. Specifically, unexpectedly high CRP reduced certainty [*b* = −0.41 (−0.609 to −0.22), *P* < 0.001], with the other categories having no/minimal impact on certainty.

Some GP comments suggested confusion as to whether CRP values were elevated or not (Supplement S4).

## Discussion

This online behavioural experiment found that striking differences in the rate of CRP requests between UK and Norway GP samples, reflecting the extent of routine use of CRP-POCT in their respective countries, did not translate to differences in the rate of antibiotics prescriptions. High CRP values increased prescriptions, while low CRP had no/minimal impact. There were too few CRP requests by the UK sample to allow strong conclusions, however, in the Norwegian sample, the data confidently show that low CRP did not reduce antibiotic prescriptions and hospitalizations. This finding contrasts with evidence from some clinical trials and a Cochrane review concluding that CRP-POCT is (likely) effective at reducing antibiotic prescribing.^5,10,14,21,22^

The mechanism underlying CRP’s effectiveness as an antimicrobial stewardship tool is unknown. It has been suggested that low CRP values, encountered in around 90% of primary care consultations, would dissuade from prescribing a clinician who is unsure and/or reassure a patient who expects antibiotics.^23^ The suggestion that CRP is useful mainly when GPs are unsure about the need for antibiotics^24^ was not supported in our study. Although higher uncertainty led to more CRP requests, the CRP result did not significantly increase certainty. A prospective point-prevalence audit of almost 5000 primary care consultations for respiratory tract infections (RTIs) across 18 European countries similarly found that GPs’ antibiotic prescribing confidence was no higher at the end of the consultation when point-of-care testing, including CRP, had been used.^13^ It is also worth noting that GPs in this audit rarely indicated uncertainty about their antibiotics prescribing decision,^13^ which further limits the scope of POCTs in calibrating uncertainty.

Presently, the CRP result carried weight and changed decisions mainly when CRP was high. High CRP reduced certainty when it conflicted with the GP’s clinical impression and minimally increased certainty when it supported expectations. In contrast, a low CRP result had no impact on certainty, whether it was supportive or not. A recent study not only predicted this asymmetry but also suggested a potential explanation: ‘Some clinicians might be more inclined to set aside a result when the test result is negative or shows low CRP concentrations, whereas a high CRP result might be followed more readily, possibly reflecting a degree of risk aversion’.^25^ Their qualitative process evaluation of the PRUDENCE trial showed how clinicians might rationalize a negative POCT result, questioning the test’s sensitivity, their own testing technique or the timing of testing relative to symptom onset, rather than revising their clinical impression. The fact that CRP cannot distinguish between viral and bacterial infections^23,26^ may partly explain why the GP’s clinical impression sometimes weighed more heavily than the CRP result.

Relatedly, the level of certainty about the need for antibiotics at the start of the scenarios was strongly associated with CRP requests, antibiotics prescriptions and certainty at the end. That is, the GP’s initial leaning based on the patient’s age, sex, medical history, risk factors and presenting complaint appeared to drive the process and determine final decisions. The strong impact of the initial clinical impression is a common finding in studies of diagnostic reasoning.^27,28^ The psychological phenomenon of ‘predecisional information distortion’ offers an explanation: clinicians begin to formulate a judgement early, with little information, which colours their interpretation of subsequent information.^29,30^ New information is not assessed independently from the current belief but can be distorted to support it.

There is some evidence that the introduction of CRP-POCT for acute respiratory tract infections in primary care can lead to its overuse without a concomitant reduction in prescribing. A study in Danish general practice found that over half of the patients with RTI symptoms were CRP-tested and, when values exceeded 40 mg/L, most were prescribed antibiotics.^31^ Anecdotal evidence also suggests that in Scandinavia, the test may even be performed prior to clinical assessment.^31^ If CRP-POCT is introduced in routine UK primary care, it may give patients the impression that it is required in every RTI consultation. This can increase demand for CRP testing, requests for further appointments, costs, disagreements between patient and clinician, and demand for antibiotics when CRP results are borderline.

A recent online experiment that used vignettes of hypothetical consultations between GPs and RTI patients found that explaining to respondents that the patient’s symptoms were viral and that antibiotics only work for bacterial infections reduced perceived need for antibiotics compared with controls.^32^ Adding the GP’s assertion that ‘there is no inflammation in your body’ based on CRP testing was even more effective—though such an assertion is unlikely given that viral infections necessitate a degree of inflammation. Clinical trials provide further evidence for the effectiveness of good communication strategies.^33,34^ For example, a six-country cluster randomized trial found that training GPs in communication skills is more effective than CRP in reducing antibiotic prescribing, especially in the long term.^33^

### Limitations

The CRP values used in the scenarios may have caused some confusion to GPs, as evidenced by some free-text comments (Supplement S4). CRP is considered low at <20 mg/L, intermediate at 20–99 mg/L and high at >99 mg/L.^35^ Thus, the borderline low values in two ‘low’ scenario versions (David with 18 mg/L and William with 19 mg/L) and the moderately raised CRP in the ‘low’ version of Carol (32 mg/L) could have caused confusion to GPs. However, these are plausible values when an infectious disease process is underway. Had we chosen very low and very high CRP values, we might have significantly influenced both decisions and certainty but at a cost to realism.

Scenarios were presented online in written form, precluding any interaction with patients, whose expectations for antibiotics can exert a strong influence on GPs’ decisions.^36,37^

### Conclusions

The present study finds no evidence that CRP-POCT would curb antibiotic overprescribing in UK primary care. A low CRP value is unlikely to reassure, while a high CRP value will increase prescribing, even if it contradicts the GP’s clinical assessment. In situations of uncertainty, CRP-POCT may not bring the desired clarity. Given that patients’ expectations for antibiotics and GPs’ perceptions of those expectations drive antibiotics prescribing to a large extent,^37–39^ scalable interventions that target these should be developed and tested in realistic settings, alongside the development of more accurate POCTs.

## Supplementary Material

dlag209_Supplementary_Data

## Data Availability

The data are available in a public, open-access repository. Dataset: https://osf.io/6uctp/files/xuc8e?view_only=21981583e4d844e49eafffef97b9f256. Key: https://osf.io/6uctp/files/w9u4c?view_only=21981583e4d844e49eafffef97b9f256.

## Supplementary data

Tables S1 and S2 are available as Supplementary data at *JAC-AMR* Online.
