# Supplementary material for: Is C-reactive protein the answer to antibiotics over-prescribing in primary care? A behavioural experiment in two European countries
Source: JAC Antimicrob Resist. 2026 Sep 17;8(5):dlag209. doi: 10.1093/jacamr/dlag209 (PMC13583623; doi:10.1093/jacamr/dlag209)
Supplement: Supplementary file 1 [file dlag209_supplementary_data.docx]

**Supplement 1**

**Study scenarios (English)**

**Patient name: David H**

**Age:** 35

**Sex:** male

**BMI:** 25

**Smoking history:** never smoked

**Past medical history:** no known chronic lung disease or other chronic conditions

**Medications:** no regular medications

**Presenting complaint:** cough productive of green phlegm and fever (not measured) for five days. Body feels achy with runny nose and sore throat.

**Appearance:** appears well

**Temperature:** 37.9°C

**Breathing:** comfortable, respiratory rate normal

**Oxygen saturation:** 98%

**Pulse:** 92

**Chest auscultation:** normal

**CRP:** 18 (low) or 126 (high)

**Patient name: Carole B**

**Age:** 75

**Sex:** female

**BMI:** 27

**Smoking history:** ex-smoker

**Past medical history:** known moderately severe chronic obstructive pulmonary airways disease (COPD), not requiring domiciliary oxygen. Three infective exacerbations in the last 2 years.

**Medications:** COPD inhalers

**Presenting complaint:** worsening cough with increased production of white phlegm, aching pains both sides lower ribs, and fever (highest 38.5°C) for three days. Runny nose and sore throat.

**Appearance:** appears well

**Temperature:** 37.6°C

**Breathing:** respiratory rate slightly raised (20 breaths per minute) with audible wheeze and no accessory muscle use

**Oxygen saturation:** 91% (usually 94% when well)

**Pulse:** 102

**Chest auscultation:** wheeze audible both sides of chest, good air entry throughout chest

**CRP:** 32 (low) or 75 (high)

**Patient name: William T**

**Age:** 25

**Sex:** male

**BMI:** 23

**Smoking history:** never smoked

**Past medical history:** no known chronic conditions; last consultation was for a sports injury (shoulder) 9 months ago

**Medications:** no regular medications

**Presenting complaint:** lower abdominal pain and nausea for the last 24 hours. Low grade fever (not measured). A day or two of lower right-sided abdominal pain. Possibly passing urine a little more often than usual.

**Appearance:** slightly distressed

**Temperature:** 37.8°C

**Abdominal exam:** tenderness in lower right quadrant with no abdominal guarding

**Urine dip/U-stix:** negative

**CRP:** 19 (low) or 110 (high)

**Patient name: Chloe S**

**Age:** 6

**Sex:** female

**Past medical history:** no known chronic conditions. She had a course of antibiotics for suspected UTI 1 year ago and had a quick recovery.

**Medications:** no regular medications.

**Presenting complaint:** dysuria, urgency, suprapubic pain and low back pain the last 24 hours. Maybe a little warm (temperature not measured).

**Appearance:** appears flushed

**Temperature:** 37.5°C

**Abdominal exam:** slight tenderness in the lower quadrants of the abdomen and no tenderness in the costovertebral angles

**Urine dip/U-stix:** leucocytes: 3+ (otherwise normal)

**CRP:** 10 (low) or 120 (high)

**Study scenarios (Norwegian)**

**Pasientnavn: David H.**

**Alder:** 35 
**Kjønn:** Mann
**BMI:** 25
**Røykehistorikk:** Har aldri røkt
**Sykehistorie:** Ingen kjent kronisk lungesykdom eller andre kroniske lidelser.
**Medikamenter:** Ingen faste medisiner.
**Aktuelt:** Hoste med grønt ekspektorat og feber (ikke målt) i fem dager. Kroppsverk, rennende nese og sår hals.

**Allmenntilstand:** Framstår som god.

**Temperatur:** 37,9°C

**Respirasjon:** David har ubesværet respirasjon og normal respirasjonsfrekvens.

**Oksygenmetning:** Davids oksygenmetning er 98%.

**Puls:** 92

**Auskultasjon av brystet:** Det er normale funn ved auskultasjon av brystet.

**Test av c-reaktivt protein (CRP):** 18 (lav) or 126 (høy)

**Pasientnavn: Carole B.**

**Alder:** 75  
**Kjønn:** Kvinne
**BMI:** 27
**Røykehistorikk:** Tidligere røyker.
**Sykehistorie:** Kjent moderat kronisk obstruktiv lungesykdom (kols), trenger ikke oksygenbehandling i hjemmet. Tre infeksiøse eksaserbasjoner i løpet av de siste to årene.
**Medikamenter:** Kols-inhalatorer.
**Aktuelt:** Forverret hoste med økt produksjon av hvitt slim, verkende smerter over nedre ribbein på begge sider, og feber (38,5 °C på det høyeste) i tre dager. Rennende nese og sår hals.

**Allmenntilstand:** Framstår som god.

**Temperatur:** 37,6°C

**Respirasjon:** Caroles respirasjonsfrekvens er noe økt (20/min) med hørbar piping. Det er ingen bruk av aksessoriske respirasjonsmuskler.

**Oksygenmetning:** Caroles oksygenmetning er 91 % (vanligvis 94 % når hun er frisk).

**Puls:** 102

**Auskultasjon av brystet:** Hørbare pipelyder på begge sider av brystet, god respirasjonslyd over alle lungeflater.

**Test av c-reaktivt protein (CRP):** 32 (lav) or 75 (høy)

**Pasientnavn: William T.** 
**Alder:** 25
**Kjønn:** Mann
**BMI:** 23
**Røykehistorikk:** Har aldri røkt
**Sykehistorie:** Ingen kjente kroniske lidelser; forrige legebesøk var for en sportsskade (skulder) 9 måneder tidligere.
**Medikamenter:** Ingen faste medisiner
**Aktuelt:** Smerter i nedre del av magen og kvalme de siste 24 timene. Lavgradig feber (ikke målt). Én til to dagers magesmerter nede til høyre. Vannlating muligens litt hyppigere enn vanlig.

**Allmenntilstand:** William virker noe utilpass.

**Temperatur:** 37,8°C

**Abdomen:** Ømhet i nedre høyre kvadrant, men ingen muskulær defense.

**Urinstix:** U-stix er negativ.

**Test av c-reaktivt protein (CRP):** 19 (lav) or 110 (høy)

**Pasientnavn: Chloe S.**

**Alder:** 6 
**Kjønn:** Kvinne
**Sykehistorie:** Ingen kjente kroniske lidelser. For ett år siden gjennomgikk hun en antibiotikakur for mistenkt urinveisinfeksjon og ble raskt frisk igjen.
**Medikamenter:** Ingen faste medisiner.
**Aktuelt:** Dysuri, urgency, suprapubiske smerter og korsryggsmerter de siste 24 timene. Kanskje litt varm (temperatur ikke målt).

**Allmenntilstand:** Chloe virker rødflammete i huden.

**Temperatur:** 37,5°C

**Abdomen:** Noe øm i nedre kvadranter.  Ingen ømhet over costovertebral vinkel bilateralt.

**Urinstix:** Leukocytter: 3+. Ellers normalt.

**Test av c-reaktivt protein (CRP):** 10 (lav) or 120 (høy)

**Supplement 2**

**Screenshots illustrating the study procedure**

*Screenshot 1*. Screenshot of the initial information for *Chloe* (one of the two ‘abdominal’ cases), showing patient demographics, medical history, medications, presenting complaint, and questions to participants.

*Screenshot 2*. List of items available on request in the *Chloe* vignette. Item order was randomised per GP. The initial patient information remained accessible to GPs throughout the vignette*.*

*Screenshot 3*. The screen seen by GPs who clicked on the button labelled *‘Overall appearance’* in the *Chloe* vignette.

*Screenshot 4.* The screen seen when GPs indicated that they were ready to manage the patient (or had viewed all items).

*Screenshot 5.* The screen seen after GPs had provided their management plan.

*Screenshot 6.* Complete version of the *Chloe* vignette (with low CRP), seen after GPs had completed all four cases. GPs who were assigned to view the *Chloe* with high CRP would see the same screen, but with CRP = 120. After rating their agreement with the two statements (need for antibiotics and need for hospital), participants were asked to select options for patient management (as per screenshot 4 above) and justify their management plan (as per screenshot 5 above).

**Supplement 3**

**Antibiotics prescribing and care escalation decisions by scenario and country (Time 2)**

| **Patient presentation** |  | **David**  35-year-old male with cough, sputum, sore throat, fever, aches | **Carole**  75-year-old female with COPD, cough, sputum, sore throat, fever, rib pain | **William**  25-year-old male with abdominal pain, nausea | **Chloe**  6-year-old female with abdominal pain, discomfort when urinating, urgent need to urinate |
| --- | --- | --- | --- | --- | --- |
| **% Antibiotics prescriptions (frequency)** | UK | 25 (25) | 63 (64) | 1 (1) | 79 (80) |
|  | Norway | 16 (16) | 64 (65) | 0 (0) | 74 (75) |
| **% Care escalation decisions**  **(frequency)** | UK | 1 (1) | 23 (23) | 80 (81) | 13 (13) |
|  | Norway | 1 (1) | 4 (4) | 78 (79) | 14 (14) |

**Table S1.** The denominator for the calculation of the percentages is 101, the number of times each scenario was presented in each country. “Care escalation” includes hospitalisation and/or referral to specialist services.

| UK + NORWAY | CRP requested: High | CRP requested: Low | CRP unknown (not requested) |
| --- | --- | --- | --- |
| David: 35-yo male with cough, sputum, sore throat, fever, aches  Commonest decision at Time 3: “neither” (70%, 142/202). This was true for both those who saw the high-CRP scenario version (52%, 51/98) and those who saw the low-CRP scenario version (88%, 91/104). | | | |
| Decisions (Time 2) | **- antibiotics only** (53%, 26/49)  - neither (45%, 22/49)  - escalation only (2%, 1/49) | - **neither** (88%, 35/40)  - antibiotics only (10%, 4/40)  - escalation only (2%, 1/40) | - **neither** (90%, 102/113)  - antibiotics only (10%, 11/113) |
| Carole: 75-yo female with COPD, cough, sputum, sore throat, fever, rib pain  Commonest decision at Time 3: “antibiotics only” (61%, 124/202). This was true for both those who saw the high-CRP scenario version (74%, 75/102) and those who saw the low-CRP scenario version (49%, 49/100). | | | |
| Decisions (Time 2) | **- antibiotics only** (75%, 44/59)  - neither (17%, 10/59)  - escalation only (7%, 4/59)  - both (2%, 1/59) | **- antibiotics only** (50%, 23/46)  - neither (44%, 20/46)  - escalation only (4%, 2/46)  - both (2%, 1/46) | **- antibiotics only** (58%, 56/97)  - neither (23%, 22/97)  - escalation only (16%, 15/97)  - both (4%, 4/97) |
| William: 25-yo male with abdominal pain, nausea  Commonest decision at Time 3: “escalation only” (80%, 162/202). This was true for both those who saw the high-CRP scenario version (95%, 96/101) and those who saw the low-CRP scenario version (65%, 66/101). | | | |
| Decisions (Time 2) | **- escalation only** (100%, 58/58) | **- escalation only** (54%, 37/68)  - neither (44%, 30/68)  - antibiotics only (2%, 1/68) | **- escalation only** (86%, 65/76)  - neither (14%, 11/76) |
| Chloe: 6-yo female with abdominal pain, discomfort when urinating, urgent need to urinate  Commonest decision at Time 3: “antibiotics only” (74%, 150/202). This was true for both those who saw the high-CRP scenario version (65%, 66/101) and those who saw the low-CRP scenario version (83%, 84/101). | | | |
| Decisions (Time 2) | **- antibiotics only** (60%, 31/52)  - escalation only (31%, 16/52)  - neither (6%, 3/52)  - both (4%, 2/52) | **- antibiotics only** (75%, 38/51)  - neither (22%, 11/51)  - escalation only (2%, 1/51)  - both (2%, 1/51) | **- antibiotics only** (82%, 81/99)  - neither (11%, 11/99)  - escalation only (5%, 5/99)  - both (2%, 2/99) |

**Table S2.** The most common Time 3 decision, and the Time 2 decisions presented separately per scenario and CRP status. “Neither”: neither antibiotics nor escalation. “Both”: antibiotics and escalation

**Supplement 4**

**Free-text comments – analyses and results**

We explored all free-text comments that mentioned CRP. First, one author read all UK comments and developed an initial thematic framework and coding scheme. A second author then read all Norwegian free-text comments, applying and refining the scheme. The refined scheme was then applied to all UK and Norwegian free-text responses.

Approximately a third of participants per country (34 in the UK and 28 in Norway) wrote comments that mentioned CRP. Most of those who left comments had requested CRP. We categorised perceptions of the CRP test and interpretation of the results and present them in Figure 4 by CRP status (high/low/unknown). GPs commented that they sometimes used a high CRP to justify prescribing or care escalation and a low CRP to justify not prescribing or hospitalising. Other times, the clinical impression weighed more than the CRP result, and a small number of UK GPs weren’t sure how to assess the result. A small number of GPs who did not request CRP also made comments. They attributed the lack of CRP request to the fact that they do not normally request it in their clinical practice (UK) or do not consider it necessary or useful.
